# Supplementary material for: The impact of blockchain adoption on supply chain financing and E-commerce platform dynamics
Source: PLoS One. 2026 Jan 2;21(1):e0339597. doi: 10.1371/journal.pone.0339597 (PMC12758829; doi:10.1371/journal.pone.0339597)
Supplement: S1 Data — (DOCX) [file pone.0339597.s001.docx]

Data set

We built a two-level supply chain model consisting of a financially constrained manufacturer (M) and an e-commerce platform (P) that utilizes blockchain technology. The study examines the impact of platform channel preferences and service levels across three merchant entry modes: the platform self-operated mode, Fulfillment by Platform (FBP) mode, and Sales on Platform (SOP) mode. A game-theoretic model, is constructed to integrate both bank financing and equity financing in analyzing production and service decisions following blockchain implementation, and some useful results are obtained. Readers can replicate the results of our study through the proofs in the appendix section.

In numerical analysis of this paper, the original data of parameters are: $r_{B}=0.05$, $r_{R}=0.03$, $\phi=0.12$, $\alpha_{Z}=1.5$, $\alpha_{F}=1.4$, $\alpha_{S}=1.3$, $k=1$, $p_{D}=0.55$, $c=0.01$, $\omega=0.35$,$\gamma=0.2$, $c_{b}=0.02$. When studying the impact of blockchain technology on channel preferences, in order to eliminate the influence of the spillover effects of blockchain technology, we set $\theta=1.5$. When analyzing the spillover effects of blockchain technology, in order to eliminate the influence of the impact of blockchain technology on channel preferences, we set $\mu=1.5$. In the analysis of sections 5.2 and 5.3,, we set $\theta=2$., $\mu=2$. Substituting these data to equations we obtained in the paper, the readers can redraw the figures in this paper.
