# Supplementary material for: The impact of blockchain adoption on supply chain financing and E-commerce platform dynamics
Source: PLoS One. 2026 Jan 2;21(1):e0339597. doi: 10.1371/journal.pone.0339597 (PMC12758829; doi:10.1371/journal.pone.0339597)
Supplement: Attachment — Submitted filename: Reviewer #3 Report - Second Revision.pdf [file pone.0339597.s006.pdf]

Reviewer #3 – Second Revision Decision

After carefully evaluating the revised manuscript **PONE-D-25-31648_R1**, along with the authors' detailed responses to all reviewer comments, I offer the following assessment and recommendation.

Overall Evaluation

The authors have made **substantial, comprehensive, and good-faith revisions** that directly address all major issues raised in my initial review. The paper is now **significantly clearer, more accessible, more rigorous, and more grounded** in current literature and managerial relevance.

The revision introduces major structural improvements (new sections, new tables, new management insights, new sensitivity analysis, improved writing, and updated literature). The additions show clear effort and greatly strengthen the manuscript.

Based on this revision, I find the manuscript **acceptable for publication**, conditional only on very minor editorial polishing by the journal.

Detailed Assessment

1. Clarity & Accessibility – Fully Addressed

- ✓ Authors added large comparative summary tables (Tables 3 & 4).
- ✓ Added "Proof Sketches" after each proposition for readability.
- ✓ Significantly improved structure and readability.

Outcome: Issue fully resolved.

2. Updated Literature Review – Fully Addressed

- ✓ Authors added 2022–2024 blockchain supply chain studies.
- ✓ Integrated them in the text rather than listing them.

Outcome: Issue fully resolved.

3. Model Assumptions Justification – Fully Addressed

- ✓ New subsection (Section 3.3) discusses and justifies each assumption.
- ✓ Provides robustness discussions.

Outcome: Issue fully resolved.

4. Sensitivity / Numerical Analysis – Fully Addressed

- ✓ Entire numerical analysis section rewritten and expanded.
- ✓ Includes sensitivity to c_b, γ, θ and detailed figures.

Outcome: Issue fully resolved.

5. Managerial & Policy Implications – Fully Addressed

- ✓ New “Management Insights” section with clear stakeholder-focused recommendations.

Outcome: Issue fully resolved.

6. Limitations & Future Research – Fully Addressed

- ✓ New subsections in the Conclusion.
- ✓ Clearly states model limitations and realistic future research directions.

Outcome: Issue fully resolved.

7. Sentence Structure & Grammar – Addressed

- ✓ Introduction and Literature Review rewritten for clarity.
- ✓ Professional editing completed.

Outcome: Issue resolved.

8. Terminology & Notation Consistency – Fully Addressed

- ✓ All terminology standardized.
- ✓ Centralized table of notation created.
- ✓ Appendices revised for clarity.

Outcome: Issue resolved.

Minor Remaining Comments

These are editorial rather than scientific issues and do not require further revision by authors:

- ✓ Some long paragraphs could potentially be split to improve readability, but this is optional and at the journal’s discretion.
- ✓ Ensure final formatting (figure placement, table cross-references, uniform line spacing) conforms to PLOS ONE layout guidelines.

These are not reasons for further revision.

Final Recommendation: ACCEPT WITH MINOR EDITORIAL POLISHING

The authors have satisfactorily addressed all major and minor concerns. The manuscript is now **appropriate for publication** in PLOS ONE, pending light copyediting by the journal.
