# Supplementary material for: The impact of blockchain adoption on supply chain financing and E-commerce platform dynamics
Source: PLoS One. 2026 Jan 2;21(1):e0339597. doi: 10.1371/journal.pone.0339597 (PMC12758829; doi:10.1371/journal.pone.0339597)
Supplement: Attachment — Submitted filename: Manuscript ID.docx [file pone.0339597.s003.docx]

**Manuscript ID:** PONE-D-25-31648
**Title:** The Impact of Blockchain Adoption on Supply Chain Financing and E-Commerce Platform Dynamics

Dear Editor,

Thank you for inviting me to review this manuscript. The study addresses an important and timely topic by examining blockchain adoption in dual-channel supply chains, integrating financing strategies and platform dynamics. While the research shows promise, I recommend major revisions to strengthen the manuscript before publication. Below are my specific comments:

### ****Major Concerns****

1. The manuscript has many grammatical issues. It would benefit from thorough professional English editing.

2. Literature Review lacks critical synthesis. Many citations are presented in list format without clearly identifying the research gap.

3. The realism of these assumptions and possible extensions in the limitations or discussion section should be further discussed.

4. The mathematical results are comprehensive, but no empirical data or simulation is used to validate the model or demonstrate sensitivity.

5. Figures/tables (e.g., Figure 4) are referenced but not well-integrated into the text. Their insights should be explained more clearly.

6. Highly technical appendix proofs could be summarized in the main text or moved to supplementary materials.

7. Recommendation: Include numerical simulations or illustrative graphs showing how key parameters (e.g., service sensitivity γ, blockchain cost cb, financing type) affect decisions and profits.

8. The article lacks a dedicated section translating results into actionable insights for practitioners (e.g., when platforms should offer financing or adopt blockchain).

Regards,

Reviewer.
