# Supplementary material for: The impact of blockchain adoption on supply chain financing and E-commerce platform dynamics
Source: PLoS One. 2026 Jan 2;21(1):e0339597. doi: 10.1371/journal.pone.0339597 (PMC12758829; doi:10.1371/journal.pone.0339597)
Supplement: Attachment — Submitted filename: Response to Reviewers.docx [file pone.0339597.s005.docx]

Dear Editor,

Thank you for the opportunity to revise our manuscript titled “The Impact of Blockchain Adoption on Supply Chain Financing and E-Commerce Platform Dynamics” (ID: PONE-D-25-31648). We are very grateful to you and the anonymous reviewers for the time and effort invested in providing such thorough and constructive feedback. The comments were invaluable and have guided us in significantly improving the quality, clarity, and contribution of our paper.

We have carefully considered every comment and have revised the manuscript accordingly. Major revisions include: strengthening the introduction and literature review, adding a dedicated section for model assumptions and a table of notations, moving detailed proofs to supplementary materials while adding proof sketches, and most substantially, completely overhauling the numerical analysis section and adding an entirely new section on actionable management insights.

Below, we provide a point-by-point response to the comments from Reviewers. For ease of reference, we have highlighted all major changes in the revised manuscript.

**Reviewer #1:**

Major Concerns

1. The manuscript has many grammatical issues. It would benefit from thorough professional English editing.

**Response 1**: We are very grateful for this valuable suggestion. We agree that the quality of language is critical, and we apologize that the previous version did not meet the required standard. In response to your comment, the manuscript has undergone a thorough professional English editing process. Great efforts have been made to improve the language, correcting all grammatical and stylistic issues to ensure clarity and precision. We hope the revised manuscript is now suitable for publication.

2. Literature Review lacks critical synthesis. Many citations are presented in list format without clearly identifying the research gap.

**Response 2**: We are very grateful for this constructive and insightful comment. We agree that the original literature review was more descriptive than synthetic and did not adequately highlight the research gap. Following your suggestion, we have substantially restructured and rewritten the entire Literature Review section (**Section 2**). Specifically, we have moved away from a ‘list format’ and have now organized the review around key themes and debates to provide a more critical synthesis. Crucially, and in direct response to your comment, we have added a concluding summary at the end of each subsection to clearly articulate the specific research gap. Furthermore, to enhance clarity, we have included a new table (**Table 1**) to chronologically position our work within the field. The revised section can be found on **pages 4-7** of the updated manuscript (**Please refer to the red text on lines 117-234 on pages 4-7**). We believe these extensive revisions now provide a strong, logical foundation for our study.

3. The realism of these assumptions and possible extensions in the limitations or discussion section should be further discussed.

**Response 3**: We thank the reviewer for this insightful suggestion. We agree that a discussion of our model’s assumptions and potential extensions is crucial. Accordingly, we have added two new dedicated subsections to the Conclusion: **Section 8.2** ‘Limitations’ and **Section 8.3** ‘Future Research’ (**Please refer to the red text on lines 1179-1211 on page 34**). In the ‘Limitations’ subsection, we now discuss the realism of core assumptions, such as a linear market demand function and homogeneous consumer valuation. In the ‘Future Research’ subsection, we build upon these limitations to outline extensions, such as incorporating alternative financing instruments and developing a multi-stage dynamic game model. We believe these additions significantly strengthen the paper by contextualizing our contributions.

4. The mathematical results are comprehensive, but no empirical data or simulation is used to validate the model or demonstrate sensitivity.

**Response 4**: We are very grateful for this critical and constructive feedback. We completely agree that grounding our theoretical model with real-world evidence is essential. To address this, we have made a substantial addition to the manuscript: an entirely new section, **Section 6**, dedicated to ‘Case Studies’ (**Please refer to the red text on lines 916-1080 on pages 28-32**). This new section validates and illustrates our theoretical findings through an in-depth analysis of JD.com’s “Zhizhen Chain” platform across three operational modes (Self-operated, FBP, SOP). Crucially, this section provides quantitative evidence for the mechanisms in our model, such as demonstrating a 58% reduction in service response time, a 12.3% price premium, and securing financing at rates 2.3 percentage points lower than traditional loans. By bridging our mathematical results with tangible data, we believe the manuscript’s credibility has been significantly enhanced.

5. Figures/tables (e.g., Figure 4) are referenced but not well-integrated into the text. Their insights should be explained more clearly.

**Response 5**: Thank you for pointing out the need for better integration of our figures. We have thoroughly revised the text in the Results section to address this. For each figure (including **Figures 4-8**), we now first describe what the figure shows (e.g., trends, key points) and then immediately explain what these findings imply in the context of our model and its managerial insights (**Please refer to the red text on lines 603-915 on pages 19-28**). We believe this new approach makes the insights from our figures much clearer and better woven into the overall argument.

6. Highly technical appendix proofs could be summarized in the main text or moved to supplementary materials.

**Response 6**: We sincerely thank the reviewer for this excellent suggestion on how to better present the technical proofs. We agree that improving the readability of the main text while maintaining full scientific rigor is very important.

Following your advice, we have implemented a solution that combines both of your suggestions:

(1) We have moved all the detailed mathematical proofs from the former Appendix into a separate document titled ‘**Online Supplementary Materials**’. This document will be submitted alongside the main manuscript for online publication.
(2) In the main text, immediately following each proposition, we have added a concise ‘**Proof Sketch**’ (**Please refer to the red text on lines 443-452 on page 13, lines 478-489 on page 14, and lines 513-521 on page 15**). This new paragraph briefly outlines the core logic and key steps of the proof (e.g., establishing concavity, applying first-order conditions), providing the reader with the essential intuition without getting lost in algebraic detail.

We believe this hybrid approach offers the best of both worlds: it makes the main manuscript much more streamlined and reader-friendly, while ensuring that the complete, rigorous proofs remain fully accessible to interested readers and reviewers. We are confident this change significantly improves the structure of our paper.

7. Recommendation: Include numerical simulations or illustrative graphs showing how key parameters (e.g., service sensitivity γ, blockchain cost cb, financing type) affect decisions and profits.

**Response 7**: We are sincerely grateful to the reviewer for this insightful recommendation. We fully agree that a robust numerical analysis is crucial for illustrating our model’s implications and providing actionable managerial insights. The reviewer’s comment prompted us to conduct a comprehensive overhaul of this part of our manuscript to ensure its clarity, depth, and impact.

In response to this suggestion, we have substantially revised and expanded our original Section 4, now retitled ‘Numerical Analysis’ (**Section 5**) (**Please refer to the red text on lines 603-915 on pages 19-28**). This new section goes beyond a simple comparative analysis and now presents a systematic numerical study structured into three distinct parts:

(1) Sensitivity Analysis (**Section 5.1**): We now explicitly investigate how optimal decisions and profits are affected by key parameters. For each figure, we have moved beyond mere description ('As shown in the figure...') to a more analytical narrative. We first pose an analytical question, then present the graphical evidence, and finally provide a detailed explanation of the underlying economic and strategic mechanisms that drive the observed trends (e.g., U-shaped price curves, monotonic service level changes). This analysis includes the impact of consumer preferences ($\alpha$) and blockchain spillover effects ($\theta$), which are directly related to the value perception that parameters like service sensitivity ($\gamma$) aim to capture.
(2) Blockchain Adoption Decisions (**Section 5.2**): This section numerically analyzes the strategic decision of whether to adopt blockchain technology. Through a series of profit comparison graphs (Figures 6 and 7), we identify critical thresholds and decision-reversal points, providing a clear decision-making framework for both the platform and the manufacturer under different operational modes.
(3) Financing Strategy Analysis (**Section 5.3**): This section directly responds to the reviewer's comments on 'financing types', focusing on the analysis of financing decisions. Specifically, Figure 8 visualizes the optimal financing choice (bank loan vs. equity financing) within a decision domain defined by production cost and product price. Crucially, and in direct response to the reviewer’s request concerning ‘blockchain cost $c_{b}$’, we demonstrate how this decision domain shifts as $c_{b}$varies (from 0.02 to 0.08), offering powerful visual insights into the interplay between technology cost and financial strategy.

In summary, the revised **Section 5** is now a comprehensive numerical study that not only includes the simulations requested by the reviewer but also presents them within a more structured, insightful, and managerially relevant framework. We are confident that these extensive revisions have significantly strengthened the manuscript and thoroughly addressed the reviewer’s valuable feedback.

8. The article lacks a dedicated section translating results into actionable insights for practitioners (e.g., when platforms should offer financing or adopt blockchain).

**Response 8**: We are very grateful to the reviewer for this critically important point. We agree completely that translating our theoretical results into actionable insights for practitioners is essential for the paper’s impact and contribution. The reviewer rightly pointed out that our initial manuscript was lacking a dedicated section for this purpose.

To thoroughly address this, we have added an entirely new, dedicated section titled ‘Management Insights’ (**Please refer to the red text on lines 1082-1143 on pages 32-33**). This section is designed to serve as a practical guide for key stakeholders.

Recognizing that ‘practitioners’ are not a monolithic group, we have structured this new section into three distinct subsections to provide tailored, actionable advice for:

(1) E-commerce Platform Managers: We provide clear guidance on when to adopt blockchain based on their operational mode (self-operated vs. FBP/SOP) and their partners’ financing choices.
(2) Small and Medium-sized Enterprises (Manufacturers): We offer a framework for making optimal financing decisions (bank loan vs. equity) based on their production cost structure and how blockchain adoption alters these choices.
(3) Policymakers: We propose differentiated policy recommendations, moving beyond a ‘one-size-fits-all’ approach to foster a healthy supply chain finance ecosystem.

We are confident that this new ‘Management Insights’ section now fully addresses the reviewer’s concern and significantly enhances the practical value and contribution of our research. We sincerely thank the reviewer for guiding us to make this crucial addition.

**Reviewer #2:**

I am really grateful to review this manuscript. In my opinion, this manuscript can be published once some revision is done successfully. I made one suggestion and I would like to ask your kind understanding.

This study develops a two-level supply chain model consisting of a financially constrained manufacturer (M) and an e-commerce platform (P) that utilizes blockchain technology. The study examines the impact of platform channel preferences and service levels across three merchant entry modes: the platform self-operated mode, Fulfillment by Platform (FBP) mode, and Sales on Platform (SOP) mode. A game-theoretic model, based on Stackelberg theory, is constructed to integrate both bank financing and equity financing in analyzing production and service decisions following blockchain implementation. This study explores the decision-making processes of both the e-commerce platform and the manufacturer regarding blockchain adoption and their respective financing strategies. Based on the results of this study, the adoption of blockchain increases profits for both manufacturers and platforms; however, decision variables exhibit different sensitivities depending on the entry mode. The self-operated mode offers more flexibility in service levels and pricing, while the FBP and SOP modes tend to show more stable decision dynamics. Moreover, the decision to implement blockchain is influenced by financing methods and channel preferences, with e-commerce platforms being more inclined to adopt blockchain in the self-operated mode, particularly under equity financing. Manufacturers also exhibit distinct financing preferences based on production costs, with bank financing being more advantageous at lower production costs and equity financing preferred when costs are higher. These findings provide valuable insights into how blockchain technology can impact supply chain dynamics, financing decisions, and the overall competitiveness of platform-based business models. I would argue that this is a good start.

However, it can be noted that every simulation study including this study has an issue of external validation, i.e., how to validate models with external data. In this context, I would like to ask the authors to address this issue in great detail.

**Response**: We would like to express our most sincere gratitude once again for your profound insight regarding the external validation of our study. Your comment strikes at the heart of a core challenge for theoretical modeling research and has compelled us to connect our model’s theoretical derivations with complex business realities. We fully agree with the importance of your feedback and, in response, have made a systematic and substantial addition to the manuscript.

Following your suggestion, we have conducted in-depth research and written an entirely new chapter: Section 6, “Case Studies” (**Please refer to the red text on lines 916-1080 on pages 28-32**). We believe this new chapter comprehensively addresses and resolves your concerns about external validation in a manner that is rigorous, in-depth, and data-driven. We would like to explain why we believe this addition is both sufficient and powerful:

**1. Direct Mapping of the Theoretical Framework to Business Practice:**

Instead of selecting broad or ambiguous examples, we have precisely chosen the Chinese e-commerce giant JD.com and its “ZhiZhen Chain” technology platform as our research subject. More importantly, we have perfectly mapped its business practices to the three core entry modes of our model (self-operated, FBP, and SOP) by analyzing the real-world cases of three representative brands: JD Health (self-operated), Midea (FBP), and Chow Tai Fook (SOP). This ensures that our theoretical validation is not abstract but is instead grounded in real and distinct business scenarios.

**2. Provision of Strong Quantitative Evidence, Not Just Qualitative Descriptions:**

We deeply understand the importance of “letting the data speak.” Therefore, in our case analysis, we have diligently sought out and cited specific quantitative metrics from industry white papers, market reports, and official disclosures to support our arguments. For example:

- Improved Service Efficiency: Reduction in after-sales service response time by 58%.
- Enhanced Pricing Power: Price premium for blockchain-certified products of 12.3%.
- Reduced Financing Costs: Reduction in bank interest rates by 1.8 percentage points and ABS financing rates by 2.3 percentage points.
- Synergistic Operational Efficiency: Improvement in inventory turnover rate by 32% and reduction in offline return rate by 17%.

This concrete data provides strong empirical evidence for the theoretical propositions in our model regarding service levels, pricing strategies, and financing decisions.

**3. Systematic Validation of All Core Model Mechanisms:**

Our case analysis is comprehensive, systematically examining all key decision dimensions of the model:

- Service Decisions: The efficiency gains at JD Health and the cost-benefit analysis at Chow Tai Fook corroborate the logic behind service investments in different modes.
- Pricing Decisions: The price premium achieved by JD Health and the narrowing of channel price differences for Chow Tai Fook validate the impact of blockchain on pricing power and channel competition.
- Financing Decisions: JD Health’s optimized bank loans, Midea’s joint fund (akin to equity financing), and Chow Tai Fook’s innovative ABS perfectly illustrate the preferences for bank versus equity/innovative financing under different cost and partnership structures, which aligns closely with our model’s conclusions.

**4. Selection of the Most Insightful and Appropriate Validation Method for the Current Research Stage:**

For an emerging and complex field like “blockchain + supply chain,” large-scale, clean panel data is extremely scarce at this stage, and the conditions for econometric regression are not yet mature. In this context, an in-depth, multi-dimensional, and data-supported multiple-case study is the gold standard for revealing complex causal mechanisms and validating the internal logic of a theoretical model. It is better suited than broad statistical analysis to explain the “why” and “how”—precisely the aspects that theoretical modeling research most needs to validate.

**Conclusion:**

In conclusion, we are confident that the new “Case Studies” chapter has fully, deeply, and robustly addressed your recommendations regarding external validation. It has not only compensated for the limitations of a purely theoretical model but has also, through real-world evidence, significantly enhanced the practical relevance, academic rigor, and managerial value of our research. We believe the overall quality of the manuscript has been markedly improved by this major addition.

Once again, we thank you for your invaluable feedback, which has pushed our research to a new level. We look forward to your review of the revised manuscript.

**Reviewer #3:**

MAJOR ISSUES

1.Clarity and Accessibility

The presentation is quite heavy on the math, which might make it tough for the wider PLOS ONE audience to grasp. Consider summarizing the key findings in tables and diagrams to help clarify the comparative results across different scenarios. A conceptual flow diagram illustrating the decision-making process would also be beneficial.

**Response 1**: We sincerely thank the reviewer for this crucial feedback on improving the clarity and accessibility of our paper. We completely agree that for a broad audience like that of PLOS ONE, presenting our model and results in a more intuitive and accessible manner is paramount. We have taken the reviewer’s excellent suggestions to heart and have made substantial revisions throughout the manuscript to enhance its readability.

To address the reviewer’s specific suggestions, we have made the following major changes:

(1) Two New Summary Tables for Comparative Results: As suggested, we recognized the immense value in summarizing our key findings. To provide a clear, side-by-side comparison and to specifically highlight the impact of blockchain, we have introduced two new comprehensive summary tables (**Table 3 and Table 4**, on **Pages 16 and 19**). Table 3 systematically summarizes the equilibrium solutions for all models in the absence of blockchain, while Table 4 presents the corresponding solutions when blockchain is integrated. This two-table structure allows readers to directly assess the strategic impact of introducing blockchain, directly addressing the request to “clarify the comparative results.”
(2) Introduction of ‘Proof Sketches’ to Clarify Decision Processes: Regarding the reviewer’s suggestion for a conceptual flow diagram to illustrate the decision-making process, we sought to address the underlying goal—clarifying the game’s sequential structure—in a way that integrates seamlessly with the text. To this end, we have added a ‘**Proof Sketch**’ immediately following each proposition (**Please refer to the red text on lines 443-452 on page 13, lines 478-489 on page 14, and lines 513-521 on page 15**). These sketches use clear, non-technical language to outline the core logic of the proof, including the sequence of actions and the logic behind the players’ decisions (e.g., “The platform, acting as the leader in Stage 1, anticipates the manufacturer’s reaction in Stage 2…”). We believe these narrative-based sketches effectively serve a similar purpose to a flow diagram by explaining the decision-making process, but in a manner that is more organically integrated into the paper’s flow.

Given the substantial addition of the two large summary tables and the numerous ‘Proof Sketches’, we felt that also adding a flow diagram might make the manuscript overly dense with visual aids and disrupt the main narrative. We believe our current approach, which combines comprehensive summary tables for the outcomes and clear narrative sketches for the process, strikes an effective balance between rigor and accessibility.

However, we are fully committed to making this paper as clear as possible. If, after reviewing these significant changes, the reviewer and the editor still feel that a conceptual flow diagram is essential, we would be more than happy to create and incorporate one.

We are very grateful to the reviewer for guiding these important improvements to the paper’s clarity.

2.Literature Review

While the literature review does a good job in some areas, it misses out on recent research related to blockchain-enabled supply chains from 2022 to 2024. Refresh the review to include the latest empirical and theoretical studies, particularly those focusing on blockchain adoption frameworks, cost-benefit analyses, and real-world validations in supply chain settings.

**Response 2**: We thank the reviewer for this very helpful suggestion to refresh our literature review. We agree that incorporating the most recent literature is essential for ensuring our study is timely and accurately positioned within the current academic discourse.

Following the reviewer’s advice, we have thoroughly updated our Literature Review section, specifically in **Subsection 2.3** (**Please refer to the red text on lines 217-222 on page 6**), to include recent and highly relevant studies published on blockchain-enabled supply chains.

In our revision, we specifically focused on integrating new literature that addresses the key themes the reviewer highlighted, ensuring a more current and comprehensive discussion:

(1) Cost-Benefit Analyses and Adoption Barriers: We have incorporated the work of Babaei et al. [39], which discusses the significant hurdles of high investment costs and deployment barriers. This directly addresses the ‘cost’ side of the cost-benefit analysis and the challenges within adoption frameworks that the reviewer mentioned.
(2) Value Proposition and Real-World Context: To complement this, we have added recent studies by Bhatia et al. [40] and Sharma et al. [41]. These papers highlight blockchain’s role in reducing transaction costs and improving performance in specific sectors, which speaks to the ‘benefit’ side of the analysis and provides context related to real-world validation.

These new references are not merely listed; they have been integrated into our narrative to enrich our discussion on the trade-offs of blockchain adoption. This updated review allows us to more effectively frame our own research, which models these very trade-offs (e.g., blockchain cost $c_{b}$ vs. its benefits) within a strategic financing and operational context.

We are confident that these updates have significantly strengthened our literature review, ensuring it provides a current and comprehensive foundation for our research, and we thank the reviewer for prompting this improvement.

3.Model Assumptions

Some assumptions (like the uniform distribution of consumer valuations, fixed commission rates, and external blockchain costs) need a bit more backing. Offer empirical or literature-based justifications for these assumptions, and discuss how relaxing them could impact the results.

**Response 3**: This is an excellent point, and we are grateful for the opportunity to strengthen the justification for our model’s foundations. To address this comprehensively, we have added a dedicated new subsection titled “Discussion of Key Model Assumptions” (**Section 3.3**) (**Please refer to the red text on lines 359-395 on pages 10-11**).

Within this new section, for each of the three assumptions you highlighted (uniform distribution, fixed commission rates, and exogenous blockchain costs), we now provide a detailed two-part discussion:

(1) A Justification based on established literature and common industry practice.
(2) A Robustness analysis that considers the likely impact on our qualitative findings if the assumption were to be relaxed.

This addition makes our modeling choices more transparent and provides a thoughtful discussion on the boundaries and potential extensions of our work, significantly strengthening the paper’s rigor.

We believe these comprehensive revisions have substantially improved the manuscript’s clarity, timeliness, and robustness. We once again thank you for your valuable guidance in this process and hope that the revised manuscript now meets the high standards for publication in PLOS ONE.

4.Comparative and Sensitivity Analysis

The comparative analysis currently only looks at closed-form solutions. It doesn’t explore how sensitive the results are to key parameters like blockchain cost , consumer service sensitivity (γ), and spillover effects (θ). Add sensitivity analyses or numerical simulations to evaluate the robustness of the conclusions.

**Response 4**: We sincerely thank the reviewer for this insightful suggestion. We agree that a robust comparative and sensitivity analysis is essential to validate the conclusions drawn from our analytical models. The original manuscript did not explore this sufficiently, and your feedback has prompted a significant and valuable revision.

To address this, we have completely rewritten and substantially expanded our numerical analysis in **Section 5** (now titled ‘Numerical Analysis’, **Please refer to the red text on lines 603-915 on pages 19-28**). This revised section is no longer just a validation of closed-form solutions but is now a comprehensive sensitivity analysis designed to evaluate the robustness of our key findings against changes in critical parameters.

Specifically, following your recommendation, we now investigate the sensitivity of equilibrium outcomes (such as profits, service levels, and strategic decisions) to several key parameters:

(1) Blockchain Cost ($c_{b}$): In **Subsection 5.3 (Figure 8)**, we conduct a direct sensitivity analysis on the cost of blockchain. By simulating the manufacturer’s financing decision domains under three distinct levels of blockchain cost ($c_{b}$ = 0.02, 0.05, and 0.08), we demonstrate the robustness of our financing choice framework and show how cost influences the boundaries of these strategic zones.
(2) Consumer Service Sensitivity ($\gamma$) and Spillover Effect ($\theta$): We have dedicated a full subsection (**Subsection 5.1.2**) to a detailed analysis of the blockchain spillover effect ($\theta$). This analysis explores how varying levels of this parameter—which directly enhances the perceived value of service ($\gamma$)—impact prices and service levels. This directly addresses your suggestion and confirms the robustness of our theoretical claims about the critical role of service and its cross-channel effects.
(3) Numerical Simulations for Strategic Decisions: Furthermore, **Subsections 5.2.1 and 5.2.2 (Figures 6 and 7)** provide detailed numerical simulations comparing profits with and without blockchain. This analysis goes beyond the analytical solutions to show how the decision to adopt blockchain is sensitive to consumer channel preferences, thereby validating our propositions about adoption thresholds under different conditions.

We are confident that this thoroughly revised section now provides the necessary sensitivity analysis and numerical simulations to demonstrate the robustness of our conclusions. We have moved beyond static solutions to offer a richer, more dynamic understanding of the strategic implications. We are very grateful to the reviewer for pushing us to make this significant improvement.

5.Managerial and Policy Implications

Although there are some managerial insights provided, they tend to be brief and mostly qualitative. Broaden this section with more specific, actionable implications for platform managers, SMEs, and policymakers, drawing from the model results.

**Response 5**: We are very grateful to the reviewer for this crucial feedback. You correctly pointed out that our original discussion of managerial implications was too brief and lacked specific, actionable guidance for the different stakeholders in the ecosystem.

To address this comprehensively, we have added an entirely new, dedicated section titled ‘Management Insights’ (**Section 7**, **Please refer to the red text on lines 1082-1143 on pages 32-33**). This new section is structured to provide clear, actionable, and model-driven implications for the three key parties you mentioned:

(1) For Platform Managers: We now provide specific guidance on when and how to adopt blockchain based on their operational mode. We recommend that managers prioritize blockchain integration in the self-operated mode due to its higher demonstrated ROI. For FBP and SOP modes, we propose a more nuanced, threshold-based approach contingent on the manufacturer’s financing type and consumer channel preferences, providing a clear strategic roadmap.
(2) For SMEs (Manufacturers): We offer a clear framework for financing decisions. Our insights guide manufacturers to link their choice between bank and equity financing directly to their production cost structure and the platform’s operational model. Crucially, we highlight how the introduction of blockchain and its associated costs can shift the optimal financing strategy, urging managers to consider these long-term financial implications during negotiations.
(3) For Policymakers: We have extended our implications to policymakers, urging a move away from a ‘one-size-fits-all’ approach to blockchain promotion. We recommend differentiated policies, such as subsidizing adoption in high-impact self-operated modes and establishing data standards to lower the entry barrier for SMEs in agency modes. This provides a more sophisticated and effective framework for governmental support.

We believe this new section transforms our discussion of implications from a brief overview into a robust, stakeholder-specific guide grounded directly in our analytical findings. We are confident that these broadened and more specific recommendations significantly enhance the practical relevance of our paper, and we thank you for prompting this important addition.

6.Limitations and Future Research

The current conclusion doesn’t clearly address limitations or future research opportunities. Include a dedicated subsection that discusses the model's scope and constraints, and suggest potential directions for further empirical validation and model enhancements.

**Response 6**: We are grateful to the reviewer for pointing out the need to formally address the limitations of our study and outline avenues for future research. We agree that this is an essential component of a rigorous academic paper, and our original conclusion section was incomplete in this regard.

To address this, we have restructured and significantly expanded the final section of our manuscript, now titled ‘Conclusion with Future Studies’ (**Section 8**, **Please refer to the red text on lines 1179-1211 on page 34**). This revised section is now clearly divided into three distinct subsections:

(1) Conclusion: This subsection succinctly summarizes the key theoretical findings and contributions of our paper.
(2) Limitations: As suggested, we have added a dedicated subsection to candidly discuss the model’s scope and constraints. We acknowledge the limitations stemming from our idealized assumptions, such as the linear demand function and homogeneous consumer preferences for blockchain’s benefits. We also recognize that our focus on only two financing instruments (bank and equity) represents a boundary of our current model.
(3) Future Research: Building directly on the identified limitations, we now propose several specific and promising directions for future inquiry. These include:

- Incorporating more sophisticated consumer behavior models (e.g., utility or prospect theory) to capture heterogeneous preferences.
- Expanding the analytical framework to include a wider array of innovative financing instruments like crowdfunding or ABS.
- Developing a multi-stage dynamic game model to analyze the long-term evolution and strategic adjustments of blockchain adoption and financing over time.

We are confident that this new, structured conclusion not only provides a clearer summary of our work but also offers a transparent assessment of its limitations and a rich, forward-looking agenda for future research in this domain. We thank the reviewer for this valuable guidance.

7.Sentence Length and Structure

A lot of sentences are quite lengthy and packed with multiple clauses, making them tricky to follow, especially in the Introduction and Literature Review. For instance, lines 32–45 in the Introduction throw together statistics, examples, and conceptual discussions into one long-winded sentence. This can lead to reader fatigue and makes it tough to grasp the arguments. Try breaking down those complex sentences into shorter ones, each zeroing in on a single idea.

**Response 7**: We sincerely thank the reviewer for this invaluable feedback on sentence structure and readability. We completely agree that many sentences, particularly in the Introduction and Literature Review, were overly complex and could lead to reader fatigue.

To address this, we have undertaken a comprehensive revision of these sections with the specific goal of improving clarity and flow (**Please refer to the red text on lines 2-53 on pages 2-3 and lines 117-234 on pages 4-6**). Our approach involved two key actions:

(1) Breaking Down Complex Sentences: We have systematically identified and broken down long, multi-clause sentences into shorter, more direct statements. Each new sentence is now focused on conveying a single, clear idea. For example, the lengthy sentence in the original introduction (lines 32–45), which conflated statistics and concepts, has been completely restructured into a series of concise statements that build a clearer narrative.
(2) Restructuring Paragraphs for Focus: In addition to shortening sentences, we have also restructured several paragraphs to ensure each one has a single, coherent theme. A key example is in the Introduction, where a long paragraph discussing both blockchain applications and SME financing challenges has now been split into two distinct, logically connected paragraphs. This greatly improves the flow of our argument.

We are confident that these revisions have made the manuscript significantly more accessible and readable, allowing our core arguments to be understood more easily. We are grateful to the reviewer for pushing us to make these important improvements.

8.Terminology Consistency

Terms like “self-operated mode,” “proprietary mode,” and “platform self-operated mode” are tossed around interchangeably, as are “FBP mode” and “Fulfillment by Platform.” Additionally, mathematical symbols (like $s_{M}$, $p_{P}$, $\omega$) sometimes pop up without a reminder of their meanings in later sections. This can confuse readers who aren’t familiar with the terminology. Standardize the terms and create a consistent glossary early on.

**Response 8**: Thank you for pointing out the inconsistencies in our terminology. We agree that standardized terminology is crucial for reader clarity, and we apologize for the oversight. We have thoroughly revised the manuscript to ensure all terms and symbols are used consistently.

Specifically, we have implemented the following changes throughout the entire paper:

(1) Standardized Terminology for Entry Modes: We have standardized all references to the platform’s entry modes.

- Terms such as “proprietary mode” and “platform self-operated mode” have all been replaced with the single, consistent term “self-operated mode”.
- Similarly, we now introduce “Fulfillment by Platform (FBP) mode” at its first mention and use the consistent abbreviation “FBP mode” thereafter.

(2) Centralized Definition of Mathematical Symbols: To address the potential for confusion with mathematical symbols, we have ensured that all parameters and decision variables are clearly and centrally defined in a comprehensive glossary, Table 2, in Section 3 (‘The Model’). This table serves as a consistent reference point for the reader throughout the paper. By defining all symbols upfront in this manner, we aim to maintain a smooth and uncluttered narrative in the subsequent analytical sections, in line with common practice in the field.

We are confident that these revisions have resolved the terminology inconsistencies and will provide a much clearer and more pleasant reading experience. We appreciate you bringing this important detail to our attention.

MINOR ISSUES

I. Abstract: Simplify the language for readers who aren’t specialists; also, briefly mention any limitations.

**Response** I: Thank you for your valuable feedback on the abstract. We agree that the original version was too technical and did not address the study’s limitations. We have completely rewritten the abstract to improve its clarity for a broader audience and to incorporate the requested information (**Please refer to the red text on Abstract on pages 1-2**).

The revised abstract now features:

(1) Simplified and More Accessible Language: We have restructured the abstract to begin with a broader context about blockchain’s impact on e-commerce, making it more engaging for non-specialists. The language throughout has been simplified to focus on the study’s core findings and their implications, rather than on the technical details of the model’s construction.
(2) Inclusion of Limitations: As suggested, we have added a concluding sentence that explicitly and concisely outlines the key limitations of our study, namely the assumptions of linear demand and uniform consumer response, and the scope being restricted to two financing types.

We are confident that the new abstract is now clearer, more comprehensive, and more accessible to the wider readership of the journal. We appreciate your guidance in making this important improvement.

II. Notation Consistency: Make sure all variables are clearly defined before they’re used; some symbols in the Appendices show up without any explanation.

**Response** II: Thank you for this very important and detail-oriented feedback. You are absolutely correct that every variable must be clearly defined before its use to ensure clarity and replicability. We apologize for the oversight, particularly in the Appendices.

To address this issue, we have taken two primary actions:

(1) Centralized Notation Glossary: We have double-checked that all primary model parameters and decision variables used throughout the manuscript are consistently and clearly defined in our main notation glossary, **Table 2**, located in **Section 3**. This table serves as the central reference point for all core symbols.
(2) Thorough Review of Appendices: More importantly, acting on your specific feedback, we have conducted a meticulous review of all mathematical derivations in the Appendices. We identified and have now explicitly defined all intermediate or composite variables that were introduced to simplify expressions during the proofs. Every symbol used in the Appendices is now either defined in **Table 2** or is clearly explained at its first point of use within the appendix itself.

We are confident that these revisions have resolved all issues of notation inconsistency. We are grateful for your meticulousness, which has significantly improved the clarity and rigor of our manuscript.

III. Proof Presentation: In Appendices A–C, consider adding short explanations between steps to help readers follow along more easily.

**Response III**: We sincerely thank the reviewer for this constructive suggestion to improve the presentation of our proofs. We agree that the original appendices were dense and lacked the necessary narrative guidance to help readers follow the derivations.

In response, we have thoroughly revised all appendices (now located in a separate Online Supplementary Materials file to improve the readability of the main paper). We have implemented the following key changes:

(1) Added Explanatory Transitions: We have inserted short, explanatory phrases between the mathematical steps to clarify the logic and flow of the backward induction process. This includes using signposts like “Firstly,” “Next,” and “Finally,” to guide the reader.
(2) Clarified Mathematical Rationale: We now explicitly state the logic behind key steps. For instance, when assessing concavity, we now detail the conditions for the Hessian matrix to be negative definite by referring to its principal minors, rather than just stating the final condition.
(3) Specified Mathematical Operations: We have replaced passive statements with active descriptions of the mathematical operations being performed (e.g., “Setting the first-order conditions to zero…” or “Substituting the result back into the profit function…”).

We are confident that these revisions have transformed the appendices into a clear, step-by-step guide that is much easier for readers to follow. We are grateful for your guidance, which has significantly improved the clarity and rigor of our manuscript.

IV. Word Choice and Redundancy

There are moments where the same idea is repeated with slightly different wording, like when “combining blockchain and different entry modes” pops up multiple times in the Introduction. Plus, phrases like “at present,” “therefore,” and “thus” are used a bit too often without really adding clarity. This makes the text feel a bit bloated. Cut out the redundancies and tighten up the wording.

**Response IV**: We sincerely thank the reviewer for this sharp and very helpful critique of our writing style. We agree that the original introduction suffered from redundancy and overly frequent use of transitional phrases, which made the text feel bloated.

To address this, we have conducted a thorough revision and complete rewrite of the Introduction section (**Please refer to the red text on lines 2-53 on pages 2-3**), focusing on two key areas:

(1) Eliminating Redundancy by Restructuring the Narrative: Instead of repeatedly stating the paper’s scope, we have restructured the introduction to build a logical narrative. The new version now:

- First, establishes the broad context of e-commerce growth.
- Second, introduces the twin challenges of service transparency and SME financing as distinct but related problems.
- Finally, presents our study as the logical synthesis that addresses these intersecting challenges.

This narrative structure ensures that the core theme of “integrating blockchain with entry modes and financing” is introduced only once, as the culmination of the setup, thereby eliminating the repetition you correctly identified.

(2) Tightening the Wording: We performed a careful line-edit throughout the section to remove unnecessary phrases and strengthen the language.

- We have significantly reduced the use of words like “therefore,” “thus,” and “at present,” opting for more direct and active sentence constructions.
- The overall wording has been tightened to improve flow and make the text more concise and impactful.

We are confident that the revised introduction is now much clearer, more engaging, and free of the redundancies that weakened the original version. We are very grateful for your guidance, which has substantially improved the quality of our writing.

V. Grammar and Agreement

There are a few subject-verb agreement errors and some issues with article usage, such as “the manufacturer determines , the service level” where there's a missing article or spacing. While it doesn’t majorly affect understanding, it does detract from the overall polish. A careful proofreading or professional language editing would help.

**Response V**: We are very grateful for this valuable suggestion. We agree that the quality of language is critical, and we apologize that the previous version did not meet the required standard. In response to your comment, the manuscript has undergone a thorough professional English editing process. Great efforts have been made to improve the language, correcting all grammatical and stylistic issues to ensure clarity and precision. We hope the revised manuscript is now suitable for publication.

VI. Flow and Transitions

The transitions between subtopics in the literature review can feel a bit jarring, leaving readers to piece together the logical connections on their own. This reduces the overall narrative cohesion. Adding brief linking sentences can help guide readers smoothly from one theme to the next.

**Response VI**: We sincerely thank the reviewer for this insightful feedback regarding the flow and transitions in our literature review. We agree completely that the original version lacked narrative cohesion and that the connections between subtopics were not sufficiently clear.

To address this, we have undertaken a significant revision of the entire Literature Review section (Section 2) (**Please refer to the red text on lines 117-234 on pages 4-6**). We have implemented a new, structured approach to ensure a smooth and logical flow:

At the end of each subsection, we have added a dedicated transitional paragraph that follows a clear “Summarize -> Critique -> Bridge” logic. Specifically, each of these new paragraphs:

(1) Summarizes the key contributions of the literature just reviewed.
(2) Identifies a specific research gap that remains unaddressed by that body of work.
(3) Creates a logical bridge to the next subsection by demonstrating how it begins to address the identified gap.

For example, the conclusion of the “E-commerce Platform Entry Mode” subsection now explicitly points out the need to consider service decisions in a dual-channel context, leading directly and naturally into the next topic. This structure culminates in the final subsection, where we synthesize all the identified gaps to strongly motivate the specific contributions of our own paper.

We are confident that this new structure has transformed the literature review from a series of disconnected summaries into a single, coherent argument that logically and compellingly sets the stage for our research. We are very grateful for your guidance, which has fundamentally improved the narrative strength of our manuscript.

VII. Use of Passive Voice

There’s a noticeable reliance on passive voice in the Methods and Literature Review sections (“It is assumed that…”, “It is found that…”) which can lessen engagement. While not incorrect, it can make the writing feel less direct. Opt for active voice where it makes sense, especially when discussing the authors’ own contributions.

**Response VII**: Thank you for this sharp and very helpful critique of our writing style. We agree that our original manuscript relied too heavily on the passive voice, which made the text feel less direct and engaging. Your feedback prompted us to conduct a thorough stylistic review.

Following your advice, we have carefully revised the Methods and Literature Review sections, as well as other parts of the paper, to convert passive constructions to active ones wherever it enhances clarity and impact. Our goal was to take clear ownership of our modeling assumptions and to more directly attribute findings to the correct researchers.

For instance, we have made changes such as:

(1) In the Methods Section:

- Passive (Original): “It is assumed that consumer valuation follows a uniform distribution.”
- Active (Revised): “We assume that consumer valuation follows a uniform distribution.”
- We have also consistently used “we model,” “we consider,” and “we structure” to clearly articulate our research design choices.

(2) In the Literature Review Section:

- Passive (Original): “In a study by Wang et al., it was found that platforms earn higher profits.”
- Active (Revised): “Wang et al. analyzed… and revealed that platforms earn higher profits.”

We are confident that these changes have resulted in a more direct, dynamic, and authoritative manuscript. We are very grateful for your guidance on improving our writing style, which has significantly strengthened the paper.
