# Supplementary material for: The impact of blockchain adoption on supply chain financing and E-commerce platform dynamics
Source: PLoS One. 2026 Jan 2;21(1):e0339597. doi: 10.1371/journal.pone.0339597 (PMC12758829; doi:10.1371/journal.pone.0339597)
Supplement: Attachment — Submitted filename: Reviewer Comments.pdf [file pone.0339597.s004.pdf]

Reviewer’s Comments to the Editor and Authors

Manuscript ID: PONE-D-25-31648
Title: *The Impact of Blockchain Adoption on Supply Chain Financing and E-Commerce Platform Dynamics*
Recommendation: Major Revision

General Assessment

This manuscript takes a deep dive into how adopting blockchain technology affects service levels, financing options, and pricing strategies in dual-channel e-commerce supply chains, all through a detailed Stackelberg game-theoretic model. The research question is not only timely but also highly relevant, and the mathematical framework used is quite rigorous. The different scenarios explored—platform self-operated, Fulfillment by Platform (FBP), and Sales on Platform (SOP)—are well-defined, and the findings could be valuable for both academics and managers alike.

That said, the manuscript needs quite a bit of work to improve clarity, update the literature review, and better link the model to real-world implications. Though the complexity of the mathematical derivations is a strong point, it also makes the content less accessible. Additionally, there are no robustness checks, a lack of integration with the latest related studies, and no clear statement of limitations. Overall, the manuscript is understandable, but the English could use some refinement to enhance flow, conciseness, and readability. The main issues include lengthy sentences, occasional awkward phrasing, and inconsistent technical terms. Although these issues may not fully obfuscate the idea, they do reduce clarity and professionalism in the presentation.

Major issues to address

1. Clarity and Accessibility

The presentation is quite heavy on the math, which might make it tough for the wider PLOS ONE audience to grasp. Consider summarizing the key findings in tables and diagrams to help clarify the comparative results across different scenarios. A conceptual flow diagram illustrating the decision-making process would also be beneficial.

2. Literature Review

While the literature review does a good job in some areas, it misses out on recent research related to blockchain-enabled supply chains from 2022 to 2024. Refresh the review to include the latest empirical and theoretical studies, particularly those focusing on blockchain adoption frameworks, cost-benefit analyses, and real-world validations in supply chain settings.

3. Model Assumptions

Some assumptions (like the uniform distribution of consumer valuations, fixed commission rates, and external blockchain costs) need a bit more backing. Offer empirical or literature-based justifications for these assumptions, and discuss how relaxing them could impact the results.

4. Comparative and Sensitivity Analysis

The comparative analysis currently only looks at closed-form solutions. It doesn't explore how sensitive the results are to key parameters like blockchain cost (c_b), consumer service sensitivity (γ), and spillover effects (θ). Add sensitivity analyses or numerical simulations to evaluate the robustness of the conclusions.

5. Managerial and Policy Implications

Although there are some managerial insights provided, they tend to be brief and mostly qualitative. Broaden this section with more specific, actionable implications for platform managers, SMEs, and policymakers, drawing from the model results.

6. Limitations and Future Research

The current conclusion doesn't clearly address limitations or future research opportunities. Include a dedicated subsection that discusses the model's scope and constraints, and suggest potential directions for further empirical validation and model enhancements.

7. Sentence Length and Structure

A lot of sentences are quite lengthy and packed with multiple clauses, making them tricky to follow, especially in the Introduction and Literature Review. For instance, lines 32–45 in the Introduction throw together statistics, examples, and conceptual discussions into one long-winded sentence. This can lead to reader fatigue and makes it tough to grasp the arguments. Try breaking down those complex sentences into shorter ones, each zeroing in on a single idea.

8. Terminology Consistency

Terms like "self-operated mode," "proprietary mode," and "platform self-operated mode" are tossed around interchangeably, as are "FBP mode" and "Fulfillment by Platform." Additionally, mathematical symbols (like S_M, P_P, ω) sometimes pop up without a reminder of their meanings in later sections. This can confuse readers who aren't familiar with the terminology. Standardize the terms and create a consistent glossary early on.

Minor issues to address

I. **Abstract**: Simplify the language for readers who aren't specialists; also, briefly mention any limitations.

II. **Notation Consistency**: Make sure all variables are clearly defined before they're used; some symbols in the Appendices show up without any explanation.

III. **Proof Presentation**: In Appendices A–C, consider adding short explanations between steps to help readers follow along more easily.

IV. Word Choice and Redundancy

There are moments where the same idea is repeated with slightly different wording, like when "combining blockchain and different entry modes" pops up multiple times in the Introduction. Plus, phrases like "at present," "therefore," and "thus" are used a bit too often without really adding clarity. This makes the text feel a bit bloated. Cut out the redundancies and tighten up the wording.

V. **Grammar and Agreement**

There are a few subject-verb agreement errors and some issues with article usage, such as “the manufacturer determines , the service level” where there's a missing article or spacing. While it doesn’t majorly affect understanding, it does detract from the overall polish. A careful proofreading or professional language editing would help.

VI. Flow and Transitions

The transitions between subtopics in the literature review can feel a bit jarring, leaving readers to piece together the logical connections on their own. This reduces the overall narrative cohesion. Adding brief linking sentences can help guide readers smoothly from one theme to the next.

VII. Use of Passive Voice

There’s a noticeable reliance on passive voice in the Methods and Literature Review sections (“It is assumed that…”, “It is found that…”) which can lessen engagement. While not incorrect, it can make the writing feel less direct. Opt for active voice where it makes sense, especially when discussing the authors’ own contributions.

General Summary

The manuscript has a lot of potential, but it could really use some revisions to align with PLOS ONE’s standards for clarity, relevance, and practical application. By addressing the earlier points, the authors can significantly boost both the scholarly and practical impact of their work. While the overall clarity is decent, there are a few highlighted issues—particularly with sentence length and inconsistent terminology—that need attention. It will require moderate to major editing to tackle these structural and terminology challenges. The authors might want to think about reaching out to professional academic English editing services before they resubmit.

Recommendation: Major Revision
